# The Impact of an Adapted Physical Activity Program on Bone Turnover, Physical Performance and Fear of Falling in Osteoporotic Women with Vertebral Fractures: A Quasi-Experimental Pilot Study

**DOI:** 10.3390/biomedicines10102467

**Published:** 2022-10-02

**Authors:** Francesca Maffei, Alice Masini, Sofia Marini, Angela Buffa, Nazzarena Malavolta, Pasqualino Maietta Latessa, Laura Dallolio

**Affiliations:** 1 Department for Life Quality Studies, University of Bologna, 47921 Rimini, Italy; 2 Department of Biomedical and Neuromotor Science, University of Bologna, 40126 Bologna, Italy; 3 Metropolitan Network of Rheumatology AUSL of Bologna, 40126 Bologna, Italy; 4 Departmental Program for Rheumatic Diseases, University Hospital of Sant’Orsola Malpighi, Mother Fortunata Toniolo Nursing Home, 40126 Bologna, Italy

**Keywords:** osteoporosis, vertebral fracture, bone remodeling, bone biomarkers, exercise

## Abstract

Physical activity has been indicated as a potential strategy to counteract osteoporosis (OP). This study of post-menopausal women with osteoporotic vertebral fractures investigated the effect of an adapted physical activity (APA) program on two serum bone turnover biomarkers (Bone Alkaline Phosphatase, B-ALP and C-terminal telopeptide of type 1 collagen, CTX-1), functional capacity (6-Minutes Walking Test, 6MWT), and risk and fear of falls (Tinetti and Falls Efficacy scale). The APA group (*n* = 12) performed a 1-h group session twice per week for 6 months whereas the control group (*n* = 9) was asked to maintain their current lifestyle. The exercise program did not affect the serum concentrations of B-ALP and CTX-1 biomarkers measured at the baseline and after 6 months in women of the APA group. Moreover, at the end of intervention no significant differences in serum concentrations for either biomarker was observed between the two study groups. Interestingly, when compared to the control group, women in the APA group showed significant improvement in the functional capacity measures by 6MWT (*p* = 0.037) and a decrease of the risk and fear of falls as indicated by the Tinetti test (*p* = 0.043). Based on these findings, exercise could provide new perspectives for the care and management of OP.

## 1. Introduction

Osteoporosis is a musculoskeletal disease characterized by low bone mineral density and alterations in bone microarchitecture resulting in susceptibility to bone fragility and risk of fractures [1]. About 200 million people are suffering from osteopenia and OP around the world. The increase of the elderly population and their associated risk for OP is estimated to grow threefold by 2050 [2]. In particular, OP is a major health problem among post-menopausal women. Postmenopausal women tend to be susceptible to OP due to estrogen deficiency. The reduced production of estrogen in women induces substantial bone loss [3]. It is estimated that over 40% of women with OP are likely to suffer a fracture during their lifetime. Postmenopausal women with fractures lose their ability to perform day-to-day activities and experience a decrease in quality of life. OP imposes an immense health and social cost on countries [4,5]. The economic impact is associated with hospitalisations, outpatient treatments and indirect costs due to loss of function and independence of patients [6]. Consequently, understanding the biological mechanisms underlying bone pathology is a major challenge to identify intervention strategies to counteract the related health risks of OP.

Bones have various types of cells including osteoclasts, osteoblasts, osteocytes, and bone lining cells [7]. Fundamentally, bone modelling and remodelling include osteoclast function in the removal of the bone surface and osteoblast function on precipitating new matrix in them [8,9]. This process is responsible for protecting skeleton function and for restoring fractures. Any kind of defect in bone turnover coordination would result in bone diseases including OP [10]. There are various ways to protect the skeleton from disease and resorption or at least delay the onset of correlated risk such as fractures. Several drugs like bone resorption inhibitors and bone formation stimulators are in OP treatment line-up. The most commonly used agents in Europe are: the bisphosphonates (e.g., alendronate); the inhibitors of receptor activator of nuclear factor Kappa-B ligand (RANKL) (e.g., denosumab); the raloxifene, a selective oestrogen receptor modulator; and the teriparatide, an agent derived from parathyroid hormone (PTH) [6,11]. Throughout life, an adequate dietary intake of key bone nutrients such as protein, calcium and vitamin D helps bone health and can reduce the risk of osteoporosis [6,12]. Combined calcium and vitamin D supplements in a daily dose of 0.5–1.2 g and 400–800 IU, respectively, are generally recommended in patients receiving bone pharmacological therapy, since some randomised controlled trials showed the efficacy of co-administration of the bone drug with calcium and vitamin D supplements for reducing the risk of fractures linked to OP [12,13,14,15].

**Figure 1:**
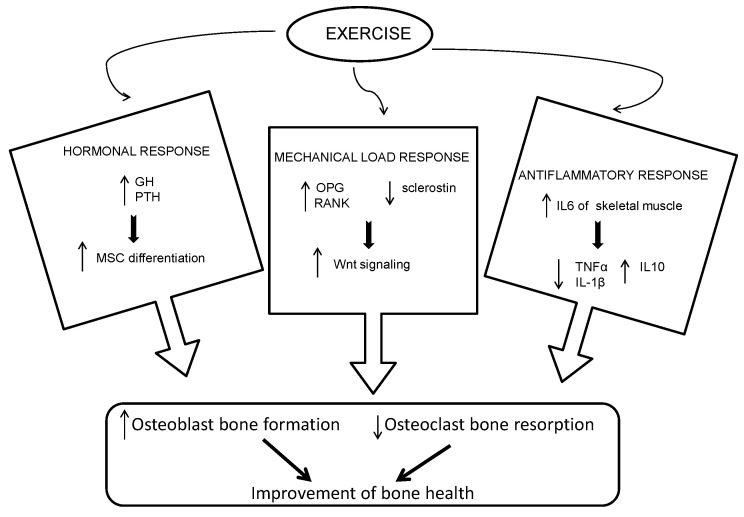
Physical exercise affects bone metabolism through various molecular and cellular pathways: stimulating the expression of some hormones; triggering the Wnt signaling pathway; activating the anti-inflammatory response. (GH: grow hormone; IL: interluchine; MSC: mesenchymal stem cell; OPG: osteoprotegerin; PTH: parathyroid hormone; RANK: receptor activator of nuclear factor Kappa-B; TNF-α: tumor necrosis factor-α).

Recent investigations have indicated that planned physical activity can effectively regulate bone turnover [9,16]. Regular, long-term, moderate-intensity physical activity would reduce bone resorption and increase bone mass in healthy and pathological individuals. Physical exercise affects bone metabolism through various molecular mechanisms and cellular reactions (Figure 1).

Exercise can determine a cycle of feedback in the hypothalamus-hypophysis-adrenal line or hypothalamus-hypophysis-gonad line, stimulating the expression of some hormones (e.g., grow hormone, PTH) which help the mesenchymal stem cell (MSC) differentiation to osteoblasts [9]. Exercise, when not excessive in terms of intensity and volume, and the following mechanical load modulates collagen synthesis during bone formation [17]. In addition, muscle tension is transferred to the bones and induces the proliferation of osteoblasts. During exercise, bone tissue deforms and the mechanosensors, located through the cells (e.g., ion channels and integrins), change their original conformation triggering several signals, including the Wnt/β-catenin signalling pathway [18]. Wnt signaling alongside induces osteoprogenitor proliferation and reduces apoptosis of mature osteoblasts; it also decreases osteoclastogenesis and the activity of osteoclast by regulating the expression of osteoprotegerin (OPG) and RANKL in osteoblasts [16,19]. Sclerostin is a protein expressed by osteocytes that plays a key role in bone metabolism since it inhibits Wnt signaling [20]. The exercise and the following mechanical load activate a molecular response that inhibits the synthesis of sclerostin and allows the activation of Wnt signalling. Afterward the bone formation increases [16]. In addition, since oxygen reactive species divert the catenin from the Wnt signaling pathway, leading to a decrease in osteoblastogenesis, the positive effect of exercise on the Wnt signaling can counteract the negative impact of oxidative stress on bone metabolism [21]. Finally, physical activity can alleviate inflammatory conditions leading to bone loss. Regular exercise activates IL-6 of skeletal muscle, which in turn inhibits the release of pro-inflammatory cytokines (TNFα and IL-1β) and induces the release of IL10, a potent anti-inflammatory molecule [16,18].

The body of evidence available evokes the need to evaluate the benefits of exercise administered to postmenopausal women with OP. To date, the dual-energy X-ray absorptiometry (DXA) for the assessment of bone mineral density (BMD) is the most widely used tool to diagnose and monitor the OP [22,23]. Some blood and urinary biomarkers have been proposed to assess the dynamics of bone metabolism and to evaluate the efficacy of therapeutic interventions for OP [24]. In addition, it has been proposed that bone markers may have a prognostic significance for OP-related fractures [6].

A few years ago, we launched the OSTEOAPA project, a quasi-experimental pilot study to assess the effectiveness and safety of an APA protocol for women with OP-related vertebral fractures [25]. The present study aimed to evaluate and discuss the effects of a 6-month exercise program on bone metabolism by the analysis of two peculiar biomarkers for the assessment of bone turnover: B-ALP, a marker of bone formation and CTX-1, a resorption reference marker [24]. Alongside, we also analysed the impact of exercise on some parameters related to OP including the fear of falling and physical performance. Our hypothesis was that a 6-month physical activity program would have a positive influence on bone turnover and physical performance and decrease the fear of falling.

## 2. Materials and Methods

### 2.1. Study Design and Participants

Our investigation is a quasi-experimental pilot study that aims to evaluate intervention without random assignment. Bone biomarker investigation was performed on a sub-group of post-menopausal women with osteoporotic vertebral fractures who participated in the OSTEOAPA project and from which a blood sample was collected before and after completing the 6-month exercise program. On the basis of the voluntary choice to perform the APA protocol, the 21 post-menopausal women involved in the present study were divided in two groups: (1) APA group; (2) control group. The Local Ethics Committee approved the study (Independent Ethics Committee, Azienda Ospedaliera di Bologna, Policlinico S. Orsola-Malpighi, ref. 143/2014/U/Sper). All individual participants included in the study gave the informed consent. 

### 2.2. Intervention

The APA group undertook a protocol of APA based on 1-h group sessions twice a week, for 6 months whereas the subjects of the control group were asked to maintain their current lifestyle. During the 6 months of the study, each patient of both groups continued to take the drugs prescribed for OP therapy. Details on study methods have been previously reported [20]. Briefly, postmenopausal women between the ages of 60 and 75 with OP, verified by dual energy X-ray absorptiometry, at least one vertebral fracture, verified by radiography, and with no significant comorbidities affecting motor or cognitive functions were recruited by the Rheumatology Section of Policlinico S.Orsola-Malpighi Institute of Bologna, Italy. The Cumulative Illness Rating Scale [26] was applied to assess the presence and the severity of other comorbidities; no significant differences were found in the two groups. No patient’s history indicated trauma as the cause of the vertebral fractures. The radiographs performed at the beginning of the study showed that the patients of both study groups had at least one grade I or II fragility vertebral fracture according to the Genant classification [27]. The vertebral fractures were asymptomatic and did not prevent the performance of the exercise program. During the study, both groups undertook pharmacological chronic therapy including antiresorptive drugs (amino bisphosphonates or denosumab) administered at the time of the OP diagnosis

The APA protocol was performed by the participants in adequately equipped gyms under the direct supervision of Kinesiologists specifically trained for the purpose by the research team. The protocol was developed over a period of 6 months and included 3 stages of progressive intensity in relation to the improvement and evolution of the abilities achieved by the participants and their feedback. In particular, the exercise intensity progression was based on the number of repetitions combined with the rate of perceived exertion, as measured by Borg Category Ratio 10 (CR-10) scale. Considering that women involved in the project had a fragility due to osteoporotic vertebral fractures, the intensity of the exercise was maintained at a light-medium level. Details of the APA protocol were previously described [25]. In summary, each session included a first stage of cardio-respiratory conditioning in order to increase body temperature and metabolism, through multi-articular exercises aimed at joint mobilization, upper and lower limb coordination, proprioception and postural education. Secondly, the main part of the session was focused on bodyweight exercises for muscular reinforcement and neuromuscular activation using isometric and dynamic bodyweight exercises to increase muscle strength and balance, without weights. Finally, the last part was predominantly mainly based on stretching exercises in an upright and supine static position for up to 30 s, breathing education, and muscle relaxation aimed at maintaining body awareness, collecting individual feedback on the session, in order to reacquire autonomy and active self-management.

### 2.3. Biomarker Assessments

Bone biomarkers are by-products of the bone remodelling process that can be assessed in serum and are indicative of bone turnover rates. In the present study, the markers used at the Rheumatology Section of Policlinico S.Orsola-Malpighi Institute of Bologna for routine clinical monitoring of patients with bone diseases have been evaluated. From the blood samples of 21 women were investigated: (1) B-ALP, a markers of bone formation [24]; (2) CTX-1, a resorption reference marker [24]. Blood samples were taken after overnight fast. Blood sampling was collected before and after the 6 months of exercise program in the two groups. Serum samples were obtained by the standard method routinely applied in the clinical laboratory. Serum B-ALP and CTX-1 markers were measured according to the protocol of iin vitro diagnostic assays provided by Immunodiagnosticsystems, PerkinElmer company, United Kingdom. The Immunoenzymatic assay IDS-iSYS Ostase^®^ BAP was used to determine the B-ALP concentration (Assay range: 1–75 µg/L- CE Marked assay). The Automated Chemiluminescence Immunoassay (CLIA) IDS-iSYS CTX-I (CrossLaps^®^) was used to determine the CTX-1 concentration (Assay range: 0.033–6.000 ng/mL). 

### 2.4. Functional Capacity, Risk and Fear of Falls Assessments

In terms of physical performance, 6-Minutes Walking Test (6-MWT) was used to assess functional exercise capacity correlated to physical fitness [28]. 

The Tinetti Scale was used to assess the motor performance aimed at balance and gait in order to identify subjects at high risk of falls [29]. The Tinetti Scale presented two different parts: balance assessment (9 items) and gait evaluation (7 items) for a total of 16 items. The evaluator assigned to each item a score ranging from 0 to 2 on the basis of the ability to perform the required actions: 0 = maximum incapacity, 2 = maximum capacity. After calculating the balance and the gait score, the evaluator obtained a total score (maximum 28) [30].

The fear of falling was assessed using the Fall Efficacy Scale-International (FES-I) questionnaire. The FES-I presented a four-level Likert scale, each of which corresponds to a score ranging from 1 (not at all worried) to 4 (very worried). The individual scores were added together to calculate a total score from 16 to 64 [31].

All measurements were collected by appropriately blinded evaluators.

### 2.5. Statistical Analysis 

Statistical analysis was performed using SPSS (Statistical Package for Social Science) (SPSS Inc., Chicago, IL, USA).

Descriptive statistics for continuous measures were reported as means and standard deviations and descriptive information for categorical variables were presented as frequency (percentages) for both APA group and Control group. Paired t-test for continuous variables was used to analyse within-group differences from baseline to follow-up. Between-groups differences over time were analysed using ANCOVA adjusted for baseline measures and age. We set a *p*-value lower than 0.05 as statistically significant (ns = non-significant).

## 3. Results

### 3.1. Study Design and Participants

The study sample included 21 women with osteoporotic vertebral fractures. As described above, the project is a quasi-experimental pilot study, so the participation in the study group was voluntary. A total of 12 women joined the APA group, and 9 women joined the control group. All 21 participants completed the study. Table 1 shows participants’ characteristics at baseline. The two study groups were similar in all characteristics except for the age. In fact, women in the APA group were older than those in the control group.

### 3.2. Bone Biomarker Assessments

In all, 21 participants’ biomarker analysis was performed at the beginning and at the end of the study. As indicated in Table 2, at the end of the follow up, no significant intra-group changes in serum concentrations were observed for either biomarker (B-ALP and CTX-1) measured at the baseline and after 6 months in the APA group and control group. Regarding the comparison between the APA group and the control group, linear regression analysis performed adjusting for baseline scores and age showed no significant differences in serum concentrations for either biomarker at the end of intervention.

### 3.3. Functional Capacity, Risk and Fear of Falls Assessments

Table 3 shows the mean scores of functional capacity and risk and fear of falls at the baseline and at the end of the study and their respective mean changes from baseline. In general, after six months of follow-up, a significant improvement in the APA group and no changes in the control group were found for the outcomes investigated. In particular, the functional capacity of the APA group assessed by 6MWT increased significantly at the end of the physical activity program. Moreover, after adjusting for age and baseline score, a group by time interaction was found, indicating a significant improvement in the APA group compared with the control group (*p* value = 0.037). A similar trend was observed for the risk of falling with a significant improvement in the Tinetti scale in the APA group comparing with controls (*p* = 0.043). The fear of falls decreased in the APA group while it slightly increased in the control group but without significant changes within and between groups.

## 4. Discussion

In the present study, we assessed the effect of a 6-month submaximal aerobic exercise program on bone turnover markers (B-ALP and CTX-1), functional capacity and risk of fall in postmenopausal women with OP.

Bone is a dynamic tissue that is highly regulated and responds to mechanical stimuli such as physical activity and exercise. Products of active osteoblasts can serve as markers of bone formation and their serum concentrations reflect osteoblast function during specific phases of bone formation [24]. In bone, B-ALP is present on the surface of osteoblasts and plays an important role in osteoid production and mineralization [32].

We did not observe a change in serum concentration of B-ALP in women who participated in the exercise program. Evidence on this topic is variable and lacking [33]. Previous studies have reported that serum B-ALP concentrations increased [34,35] or remained unchanged [36,37]. Additionally, a recent meta-analysis investigating the effectiveness of exercise in the treatment of primary OP did not report improvements of kinesiotherapy on B-ALP concentration in this group of patients [38]. These findings could be due to the temporary modulation that physical activity induces on this enzyme linked to bone formation. Experimental tests showed that B-ALP expression by osteoblast precursors transiently increased from baseline 30 min after in vitro mechanical stimulation, before returning to pre-stimulation levels 24 h later [39,40]. Significant increases in B-ALP concentrations have also been shown at the 30th and 50th minutes of cycling or at the end of the cycling session [41,42]. All changes in B-ALP returned to baseline within 20 min after exercise [43].

As is well known, CTX-1 results from osteoclast-mediated collagen degradation. Among the bone turn-over markers, CTX-1 is used to assess the bone resorption activity or to monitor the efficacy of drug therapy for OP [24,44,45]. In our study, no significant variation of CTX-1 concentration was observed in the two study-groups during the follow-up, suggesting that the training applied did not influence the production of this biomarker linked to bone resorption. This result is consistent with those of other previous studies showing no effect of exercise in circulating CTX-1 markers among women with OP [46,47,48,49].

Taking together our findings and the scientific evidence published, to date it is difficult to draw a conclusion about the effects of exercise on bone biomarkers in women with OP. This could be due to various factors related to the heterogeneity of the study design and the characteristics of exercise protocol. It is known that bone mass is the result of bone formation and resorption, which are tightly regulated by the equilibrium between endogenous/exogenous factors including physical activity. The bone preferentially responds to mechanical loads that induce high-magnitude strains at high rates or frequencies and that weight-bearing loading is important. Consequently, the effects induced by exercise on bone metabolism biomarkers depend on the type, frequency and intensity of training performed [50]. In this connection, it has been suggested that the moderate-intensity exercise for individual muscle groups could not be enough suitable to generate the requisite skeletal train to stimulate an osteogenic response [51]. Recently, has been indicated that biomarkers may be more useful for measuring effectiveness of medications than for exercises. This is because biomarkers reflect changes in overall skeletal bone loss and the whole-body rates of bone resorption and formation. Exercises are targeted to specific skeletal sites, and their effectiveness is better measured by changes in BMD at these specific sites [37]. In future perspective, it would be necessary to take into consideration even the best frequency-intensity-type-time-volume-progression combination in relation to the district activity in order to facilitate the osteogenic process in the affected tract and perhaps detect a relevant biochemical response [52].

The clinically meaningful objective of the management of OP is the prevention of fracture occurrence; thus, the reduction in fall risk should be an important focus of prevention interventions. Women with OP are at a high risk for fracture not only because of a lower bone mass, but also because of a high risk for falling. During the postmenopausal period, physical capacity decreases due to a decrease in physical activity, an increase in body weight, a decrease in muscle mass and strength, and postural changes [53,54]. Furthermore, the risk of falling increases due to hormonal changes that affect postural stability [55]. There is also a strong relationship between balance deficit and the incidence of falls [56]. Exercise is proposed to be a potential strategy to prevent falls [57]. To shed some light on this issue, another purpose of this study was to investigate the effect of the exercise program on functional capacity and risk and fear of fall in postmenopausal women with OP. Our results showed an improvement induced by exercise. Indeed, the training program applied was able to decrease the risk and fear of falls as indicated by the Tinetti test. Moreover, compared to women in the control group, women in the APA group showed significant increase in the functional capacity measures by 6MWT. These findings are in line with other studies that report the benefit of exercise to enhance the physical performance in postmenopausal women with OP [58,59]. This knowledge is important for the healthcare system because it strengthens the concept of exercise as a useful tool for reducing the risk of falling and related disabilities. Exercise can be promising also in countering the decrease in independence and quality of life that often emerges in osteoporotic patients.

Physical activity planning for individuals with low bone density and risk of fracture is closely linked to the need to identify safe training exercise that can be tolerated by participants. In addition, if exercise is proposed as a therapeutic approach for OP, adverse events should be measured. In our study we tried to address this exigency. The post-menopausal women performed the physical activity program in an adequate and equipped gym under the supervision of skilled staff who constantly evaluated and adapted the exercises according to the characteristics and abilities of the participants. Moreover, the protocol was aimed at assuring adherence and promoting motivation of participants oriented to physical activity learning and subsequent consolidation. Our results suggest that the monitored program, including multi-joint, compound movement, high-intensity progressive resistance training, balance and muscle strength exercise, was feasible and safe: no incidents or major adverse events occurred during the training sessions and all participants completed the study. These findings are in line with previous evidence that has highlighted the crucial part of the intervention aimed at reducing the risk of fractures is a scheduled program including exercises that can improve balance and muscle strength [50,60,61]. In addition, specialized staff has a pivotal role to supervise the proper execution of exercises and ensure the safety of applied protocols [50,60,61]. It is also important to keep in mind that exercise has pleiotropic positive effects on the health of postmenopausal women, including prevention of diabetes and cardio-vascular disease, reduction in cognitive decline, improvement of mood and well-being. On the basis of the above considerations, it is advisable that the prescription of the adapted exercise should be part of the optimal management of every patient with OP.

There are some strengths to this study including the unique focus on post-menopausal women with osteoporotic vertebral fracture, and the comprehensive assessment of exercise effects on bone biomarkers and parameters linked to performance capacity, risk and fear of falling. However, some limitations of the study deserve attention. Due to the small sample size and differences in terms of age between the control and the APA group, data on the efficacy of the program should be considered with caution. Furthermore, the effectiveness of the program should be assessed in a future randomized controlled study with a larger number of patients balanced for ages.

## 5. Conclusions

On the basis of our findings and limitations of the study, it is possible to posit some comments. A supervised, twice-weekly exercise intervention seemed to be useful for enhancing functional performance and reducing the risk of fall in postmenopausal women with OP and vertebral fracture. Further, no fractures or other serious injuries were sustained by any participant in our study, suggesting that the training for balance, coordination, endurance, and stretching do not pose a significant risk for postmenopausal women with OP when closely supervised by skilled personnel. Our data, examined alongside the available scientific evidence, do not allow a comprehensive conclusion on the effects of exercise on bone biomarkers. Future studies of a large population of women with OP are required to address this topic. Overall, our study suggests potential contexts for prescribing exercise as a future therapeutic option for the management of OP in postmenopausal women with vertebral fractures. The availability of information on the safety and effectiveness of training protocols will help the health system to make rational choices based on scientific evidence to organize feasible and efficient exercise interventions.

## Figures and Tables

**Table 1:** Baseline characteristics of the participants.

Variable	APA Group *n* = 12	Control Group *n* = 9	*p* Value
Age (years)	66.67 ± 1.24	51.50 ± 3.28	<0.01
Normal weight	6 (50%)	7 (77.8%)	ns
Currently smoking	0	1 (8.3%)	ns
Number of vertebral fractures	1.83 ± 1.19	2.22 ± 1.20	ns

Note: ns = not significant.

**Table 2:** Bone biomarker measured in the two study-groups at baseline, follow-up, and change at 6 months.

APA Group *n* = 12					Control Group *n* = 9				
Variable	Baseline Mean ± sd	Follow-Up Mean ± sd	Change Mean ± sd	Within Group *p* Value	Baseline Mean ± sd	Follow-Up Mean ± sd	Change Mean ± sd	Within Group *p* Value	Between Groups ^a^ *p* Value
B-ALP (µg/L)	23.10 ± 11.70	23.76 ± 12.04	0.66 ± 14.46	ns	15.13 ± 1.96	13.53 ± 3.72	−1.60 ± 5.37	ns	ns
CTX-1 (ng/mL)	0.34 ± 0.34	0.25 ± 0.32	−0.09 ± 0.42	ns	0.29 ± 0.30	0.09 ± 0.07	−0.19 ± 0.32	ns	ns

^a^ Changes in measures between baseline and follow-up are compared using linear regression with correction for baseline scores and age of the analyzed variable. Note: ns = not significant.

**Table 3:** Functional Capacity, Risk and Fear of Falls Assessments measured in the two study -groups at baseline, follow-up, and change at 6 months. Outcome measures at baseline, follow-up, and change at 6 months.

APA Group *n* = 12					Control Group *n* = 9				
Variable	Baseline Mean ± sd	Follow-Up Mean ± sd	Change Mean ± sd	Within Group *p* Value	Baseline Mean ± sd	Follow-Up Mean ± sd	Change Mean ± sd	Within Group *p* Value	Between Groups ^a^ *p* Value
Functional Capacity									
6MWT	373.01 ± 78.26	430.74 ± 58.97	57.72 ± 45.07	0.001	415.21 ± 66.68	412.29 ± 74.37	−2.92 ± 56.15	ns	0.005
Risk and Fear of Falls									
Tinetti	23.75 ± 6.35	27.25 ± 0.97	3.50 ± 6.14	ns	26.11 ± 3.55	25.44 ± 3.54	−0.67 ± 2.29	ns	0.000
FES-I	29.42 ± 9.24	26.83 ± 8.23	−2.58 ± 7.08	ns	21.44 ± 5.43	21.56 ± 4.61	0.11 ± 1.76	ns	ns

^a^ Changes in measures between baseline and follow-up are compared using linear regression with correction for baseline scores and age of the analyzed variable. Note: ns = not significant.

## Data Availability

The data presented in this study are available on request from the corresponding author: sofia.marini2@unibo.it.
